# Decision aids to prepare patients for shared decision making: Two randomized controlled experiments on the impact of awareness of preference‐sensitivity and personal motives

**DOI:** 10.1111/hex.13159

**Published:** 2021-01-31

**Authors:** Simone Korger, Marie Eggeling, Ulrike Cress, Joachim Kimmerle, Martina Bientzle

**Affiliations:** ^1^ Knowledge Construction Lab Leibniz‐Institut fuer Wissensmedien Tuebingen Germany; ^2^ Department of Psychology University of Tuebingen Tuebingen Germany

**Keywords:** decision aids, patient experiences, personal motives, preference‐sensitivity, Shared decision making

## Abstract

**Objective:**

To participate in shared decision making (SDM), patients need to understand their options and develop trust in their own decision‐making abilities. Two experiments investigated the potential of decision aids (DAs) in preparing patients for SDM by raising awareness of preference‐sensitivity (Study 1) and showing possible personal motives for decision making (Study 2) in addition to providing information about the treatment options.

**Methods:**

Participants (Study 1: N = 117; Study 2: N = 217) were put into two scenarios (Study 1: cruciate ligament rupture; Study 2: contraception), watched a consultation video and were randomized into one of three groups where they received additional information in the form of (a) narrative patient testimonials; (b) non‐narrative decision strategies; and (c) an unrelated text (control group).

**Results:**

Participants who viewed the patient testimonials or decision strategies felt better prepared for a decision (Study 1: *P *< .001, $\eta_{P}^{2}$ = 0.43; Study 2: *P *< .001, $\eta_{P}^{2}$ = 0.57) and evaluated the decision‐making process more positively (Study 2: *P *< .001, $\eta_{P}^{2}$ = 0.13) than participants in the control condition. Decision certainty (Study 1: *P *< .001, $\eta_{P}^{2}$ = 0.05) and satisfaction (Study 1: *P *< .001, $\eta_{P}^{2}$ = 0.11; Study 2: *P *= .003, *d* = 0.29) were higher across all conditions after watching the consultation video, and certainty and satisfaction were lower in the control condition (Study 2: *P *< .001, $\eta_{P}^{2}$ = 0.05).

**Discussion:**

Decision aids that explain preference‐sensitivity and personal motives can be beneficial for improving people's feelings of being prepared and their perception of the decision‐making process. To reach decision certainty and satisfaction, being well informed of one's options is particularly relevant. We discuss the implications of our findings for future research and the design of DAs.

## 1 INTRODUCTION

The question as to how shared decision making (SDM) between clinicians and patients can be improved is frequently asked in patient‐education research. SDM is an approach to reaching medical decisions ‘where clinicians and patients make decisions together using the best available evidence, where patients are encouraged to consider available screening, treatment, or management options and the likely benefits and harms of each’.^1^ Including patients in medical decisions is particularly important in preference‐sensitive situations, where there is no scientific evidence for the superiority of one treatment option.^2^ In these situations, the decision should depend on individual circumstances, values and preferences of the patient.^3^, ^4^ Most patients wish to be included in medical decisions and prefer SDM to paternalistic or purely autonomous approaches.^5^, ^6^ Using SDM results more often in decisions that fit patients’ preferences and has also been found to have positive effects on several outcomes, like satisfaction,^7^, ^8^, ^9^ patient‐physician relationship,^10^ treatment co‐operation and compliance.^10^, ^11^ However, there are reasons why SDM is often challenging for both physicians and patients which consequently discourage implementation in everyday clinical practice.

A major difficulty for successful SDM is the fact that most patients have little medical background knowledge. Gaining trustworthy knowledge about their diagnosis and treatment options for making an informed decision^12^, ^13^ thus becomes a big challenge, and many patients remember little about the treatment options after their consultation with a physician.^14^ A popular approach to deal with this problem is the application of *decision aids*.^15^, ^16^, ^17^, ^18^ Decision aids (DAs) are evidence‐based tools that are developed to support patients in making choices among health‐care options,^19^ complementary to personal consultations. They have been found to have positive effects on knowledge gain,^19^, ^20^, ^21^ satisfaction with the decision process^20^, ^21^ as well as on active participation in decision making and awareness of personal values^19^ in different medical fields.

Moreover, providing information alone is not enough to enable successful participation in medical decision making.^22^, ^23^, ^24^ Many patients tend to underestimate the importance of their personal preferences in decision making. Joseph‐Williams et al^22^ concluded that research should therefore examine methods that enable patients to recognize this importance and prepare them for SDM. In their theory, Waldron et al^25^ proposed that when patients are ‘able to express their preferences and values through the implementation of SDM, then they experience higher confidence in their ability to participate in SDM, resulting in higher levels of SDM engagement’ (p. 12). While most DAs aim to impart medical knowledge, they should also include additional material for decision support.^26^ The studies presented here address this research gap.

### 1.1 Decision support material

Two experimental studies investigated the potential of DAs that aim to prepare patients for and support SDM, in addition to being thoroughly informed about the treatment options. As many patients find it hard to grasp that their personal preferences matter in a medical decision, one way to prepare them for SDM is to explain the concept of *preference‐sensitivity* better. But even if patients have understood that they themselves and the physician involved should be more sensitive to their personal preferences when medical decisions are made, it is still challenging for them to figure out what is particularly important to them.^27^, ^28^ So, for patients who have already understood the preference‐sensitivity of the situation, the next step is to make them aware of potential *personal motives* in a decision. This approach aims at giving them a clear idea of what their decision might be based on. Obviously, in preference‐sensitive decision situations, this should be done in a way that does not manipulate patients towards one option.

### 1.2 Narrative and non‐narrative formats

There are different ways to explain the concept of preference‐sensitivity and make people aware of different motives. In the studies presented here, we compared two different kinds of formats: narrative patient testimonials and non‐narrative decision strategies.

Many DAs include narrative patient testimonials,^29^, ^30^, ^31^ as patients perceive personal experiences of others combined with factual information to be very helpful for decision making.^32^ Narrative formats have the advantage that they are vivid, easy to understand, and not abstract, making it easier for patients to comprehend and remember the information they contain.^33^, ^34^ They arouse interest and can support patients both in understanding their role in the decision‐making process and in clarifying their personal preferences. When using narratives in DAs, one needs to be careful, however, because reading about the experiences of others can bias decision making by triggering heuristic thinking.^34^, ^35^, ^36^, ^37^ According to Shaffer et al,^29^, ^38^ patient testimonials should focus on the decision process rather than on the outcome of this process.

Information regarding preference‐sensitivity and motives can also be presented in a non‐narrative format. Strategies for recognizing preference‐sensitivity or for becoming aware of personal motives may be just listed without any personal context. While a non‐narrative format appears to be less vivid and more abstract than narrative testimonials, it remains unclear which format is more helpful for people faced with a medical decision. In both studies presented here, we investigated the impact of narrative patient testimonials compared to non‐narrative decision strategies.

### 1.3 Research questions and hypotheses

In order to understand the potential of elements in DAs that prepare patients for SDM and are given in addition to detailed and balanced information about the options, we conducted two experimental studies. We addressed two aspects that may support patients in SDM: explaining the concept of preference‐sensitivity (Study 1) and presenting personal motives (Study 2). Understanding preference‐sensitivity is necessary to comprehend the concept of SDM, whereas becoming aware of possible motives underlying a decision implies that patients have already understood the concept of SDM and have started to consider their treatment options more closely. While both approaches may be helpful to prepare patients for SDM, they address different steps in the decision‐making process.

In both studies, participants received DAs that consisted of a video about a consultation with a physician and additional material. The additional material differed between the conditions and was either (a) narrative patient testimonials, (b) decision strategies or (c) a text about an unrelated topic (control condition). Both studies used hypothetical scenarios, where participants were faced with a choice regarding a medical decision. In Study 1 (additional material about preference‐sensitivity), we chose the scenario of suffering from a cruciate ligament rupture, a sports injury that can be treated either surgically or with intense physiotherapy. Neither treatment has been found to be clearly superior,^39^, ^40^ so patients in this situation were faced with an individual, preference‐sensitive decision. In Study 2 (additional material about personal motives), we used the scenario of thinking about switching from an oral contraceptive pill to an intrauterine device (IUD) with copper. A decision about a contraceptive method is preference‐sensitive, as there are many equally effective options, and women are aware of that.^41^, ^42^ Nevertheless, many women seek consultation when making this decision.^43^ This scenario allowed us to examine the potential benefits of being presented possible motives underlying a decision.

For the selection of our outcome variables, we built on Elwyn and Miron‐Shatz^4^ who suggested that the evaluation of SDM should focus on patients’ personal perceptions of being well informed, certain about their choice, and satisfied with the decision‐making process, rather than on outcomes of the decision. In both studies, we measured participants’ preparation for decision making, their decisional conflict and their evaluation of the decision process. In Study 1, we also measured participants’ control preferences (ie the amount of control one wants to assume in the decision in terms of partly handing over decision‐making powers to a physician). In Study 2, the control preferences were not included, because women demand to have personal control of their decision in the choice of a contraceptive method.^44^

We hypothesized a positive impact of reading an additional element (narrative patient testimonial or decision strategies) compared to the control condition. Previous research suggests that encouragement to participate in the decision‐making process as well as support regarding the formation of personal preferences may be helpful in addition to factual information.^22^, ^45^, ^46^ In particular, we expected that participants who viewed a DA with such additional material would feel better prepared for the decision (H1a), show a stronger increase in decision certainty (confidence that the decision is right for them) and satisfaction regarding the decision (H2a), and evaluate the decision process more positively (H3a) than participants in the control condition. In Study 1, we also expected them to prefer a more active role in the decision‐making process (control preferences; H4a).

We also hypothesized that the narrative testimonials would be more effective than the non‐narrative decision strategies. Former research has shown that narratives in DAs can support patients in understanding their role in the decision‐making process and in clarifying their personal preferences.^29^, ^32^, ^38^, ^47^ Consequently, we expected that participants in the narrative condition would feel better prepared for the decision (H1b), show a stronger increase in decision certainty and satisfaction (H2b), and evaluate the decision process more positively (H3b) than participants in the non‐narrative condition. In Study 1, we also expected them to prefer a more active role in decision making (H4b).

In Study 2, as an exploratory research question, we measured participants’ individual motives to examine whether the DAs resulted in changes of what they perceived as personally important and whether there were differences between the conditions.

## 2 MATERIALS AND METHODS

### 2.1 Participants

**Figure 1:**
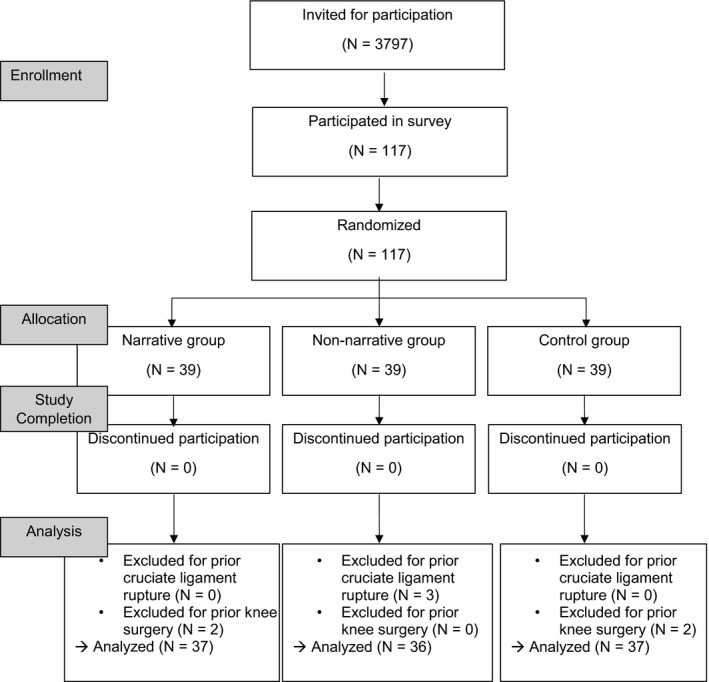
Sampling procedure of Study 1

Participants for Study 1 were recruited from the participant database of the Leibniz‐Institut fuer Wissensmedien. Registration in this database is voluntary and open for everyone. In order to include only participants with little prior knowledge about the topic, we did not invite any medical or sports students, or any people working in a medical profession. 117 laypeople participated in the laboratory experiment, and seven participants were later excluded because they had suffered from a cruciate ligament rupture or a similar knee injury before (Figure 1). In the end, we analysed the data of N = 110 participants (gender: 84 female, 25 male, 1 diverse; age: *M* = 23.43 years old*,* SD* = *2.96). They all provided written informed consent. Participation in this study took 45‐60 minutes and was compensated with 8 Euros.

Participants for Study 2 were recruited via the e‐mail distribution list of the University of Tuebingen. Participation in the study took place online, was voluntary, and all participants gave written informed consent.

**Figure 2:**
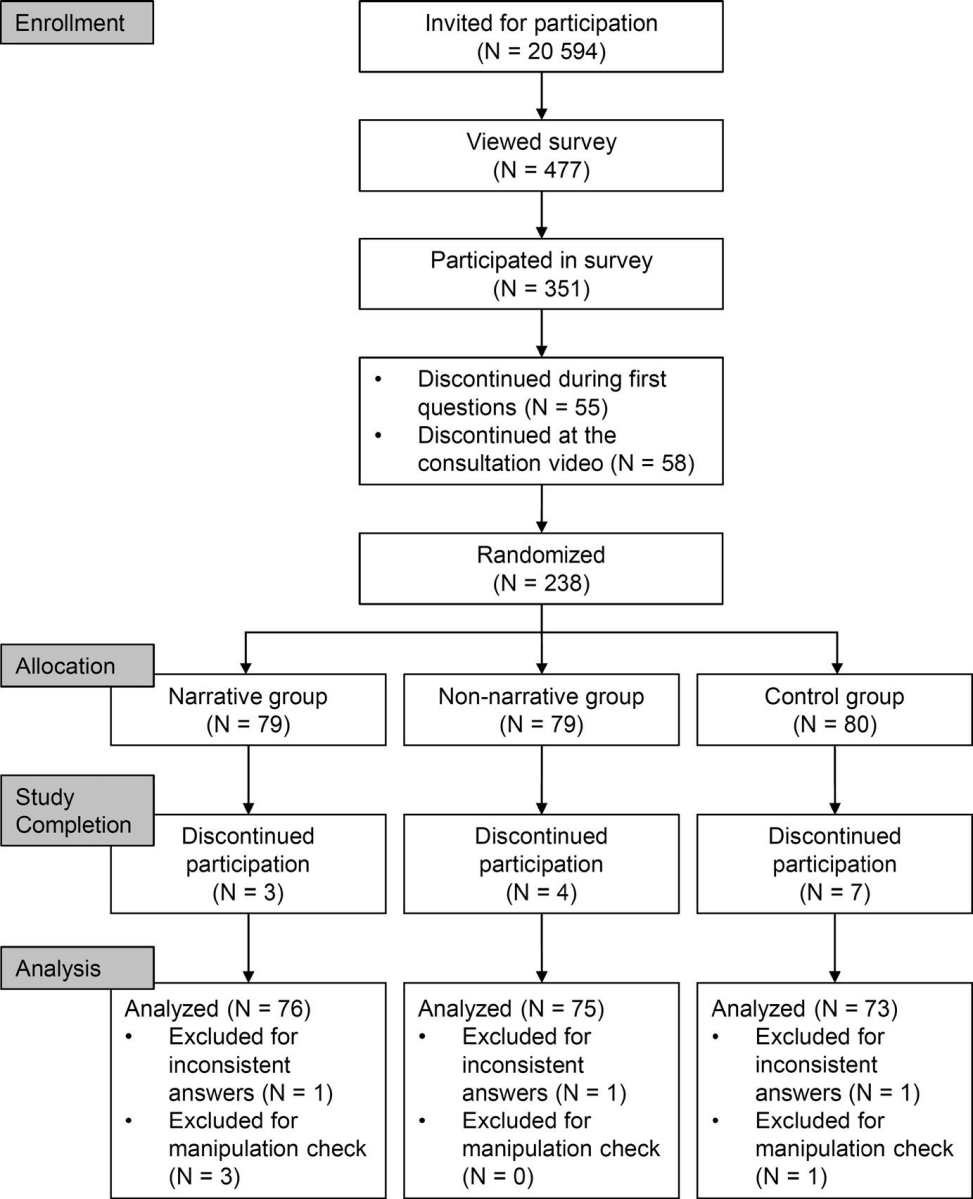
Sampling procedure of Study 2

Since a representative survey in Germany found that 77% of the young women surveyed had already sought advice from their gynaecologist regarding contraception,^43^ and many women use the oral contraceptive pill (18‐29 years: 56%),^48^ we invited only women between 18 and 35 years old who were currently taking an oral contraceptive pill. This allowed us to keep the sample equivalent and to test our hypotheses using a decision situation that frequently occurs and is relevant. N = 217 participants (*M* = 23.15 years, *SD* = 2.95) finished the online questionnaires and passed the control question, where they indicated which kind of additional material they had read (Figure 2). Participants with inconsistent answering patterns were excluded (eg one participant indicated that she was 18 years old and had used the contraceptive pill for 18 years). Participation in this study took 20‐30 minutes, and as compensation participants had the opportunity to take part in a raffle to win vouchers for an online store.

### 2.2 Procedure

In both studies, all instructions and questionnaires were presented to the participants on a computer screen. After reading the study description, participants answered demographic questions, read a general information text, and indicated their prior experiences with cruciate ligament injuries or contraception, respectively, in order to examine potential prior differences (see Measures for details).

**Figure 3:**
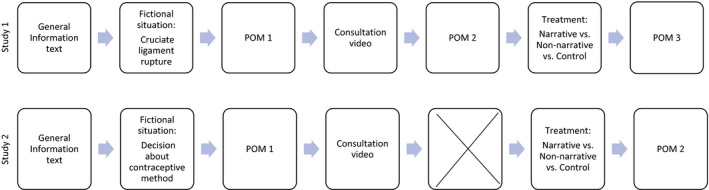
Procedure of Study 1 and Study 2

Participants then read the fictional situation (Appendix S1) and responded to questionnaires (point of measurement 1; POM 1). Afterwards, they watched a video showing a consultation with a physician/a gynaecologist. Only in Study 1 was there a measurement following the video (POM 2). This decision was made to investigate explicitly the impact of the different components of the DAs. In Study 2, we did not use POM 2 due to the timing constraints of the online study. Subsequently, participants were randomly assigned to one of three conditions and either read (a) two narrative patient testimonials, (b) non‐narrative decision strategies or (c) an unrelated text (control condition). Then, participants responded once again to questionnaires (Figure 3). To ensure that participants actually watched the video and read the texts, we included timers in the survey so that they could only continue to the next page after an appropriate time. To avoid missing data, participants were forced to respond to all of the questions before they could proceed with the survey.

### 2.3 Materials

#### 2.3.1 General information text

In Study 1, the general information text consisted of 504 words and contained five pictures. It explained the location and function of the cruciate ligament, consequences of an injury, and treatment options (surgery or intense physiotherapy), all in a neutral way. It stated that in medical research neither treatment had been found clearly better than the other (Appendix S2).

In Study 2, the general information text provided basic information about the copper IUD and how it functions (47 words) including a picture of the location of the copper IUD in the uterus. On the next page, information about the security of different contraceptive methods was given (103 words; Appendix S3).

#### 2.3.2 Consultation videos

In Study 1, the consultation video took 07:30 minutes and showed a physician (portrayed by a white male actor in his late 30s) head‐on. The video had already been used in another study^28^; the content was based on Elwyn et al’s^45^, ^49^ SDM model of making patients aware of their choices and comparing the alternatives (the script of the video is shown in Appendix S4).

In Study 2, the consultation video took 4:53 minutes and showed an interaction between a young female patient asking for information about the copper IUD and a female gynaecologist. This video had also already been used in a prior study.^41^ The gynaecologist was shown head‐on, allowing participants to put themselves in the patient's position, and was portrayed by a white actress in her 40s (Appendix S5).

#### 2.3.3 Patient testimonials and decision strategies

In both studies, the patient testimonials and the decision strategies were presented as content in an online portal, where patients could get information about a cruciate ligament rupture/about contraceptive methods and read about others’ experiences. We created the material after an intensive literature search of patients’ needs and experiences^50^ as well as suggestions from previous research.^22^, ^29^ The material standardized the given information across the conditions and kept the content in the narrative condition and in the non‐narrative condition parallel: The decision strategies and motives were the same as the ones mentioned in the narratives, but without any personal experiential information. The texts in the control condition were unrelated to the medical decision and dealt with the topic of migrating birds (Study 1) or potatoes (Study 2).

Following Shaffer et al,^29^, ^38^ the testimonials focused on the decision process and did not mention the outcome of the decision to avoid persuasive effects. To ensure that there was no persuasive effect, we asked participants which treatment they preferred and found no significant differences. In Study 1, the testimonials consisted of 320 and 317 words, respectively, and were depicted as having been posted by one male and one female patient. The alleged patients reported their experience with learning to understand preference‐sensitivity. The decision strategies for the non‐narrative condition in Study 1 consisted of six key strategies (112 words total) one could use for decision making in a preference‐sensitive situation (Appendix S6).

In Study 2, the fictional patient testimonials consisted of 329 and 327 words, respectively, and were depicted as having been posted by two females who had been taking the oral contraceptive pill in the past. The alleged authors described their thoughts in terms of their personal motives while considering changing their contraceptive method from the contraceptive pill to the IUD. The decision strategies for the non‐narrative DA condition (410 words) included all the motives that were mentioned in the narratives, but without any individual references (Appendix S7).

### 2.4 Measures

*Preparation for decision making* was measured with 10 items on seven‐point scales^51^ at the final POM (Cronbach's *α* = 0.96‐0.97).

*Decision evaluation* was measured with 15 items on seven‐point scales using the Decision Evaluation Scales^52^ at the final POM (Cronbach's *α* = 0.89‐0.91).

*Decision certainty* and *satisfaction regarding the decision* were captured on seven‐point scales using the sub‐scales ‘decision uncertainty’ (three items; Cronbach's *α* = 0.86‐0.92) and ‘perceived effective decision making’ (four items; Cronbach's *α* = 0.86‐0.90) from the Decisional Conflict Scale.^53^ In Study 1, certainty and satisfaction were assessed at all three POMs, in Study 2 at both POMs.

In Study 1, *control preferences* were assessed using the Control Preferences Scale^54^ at all POMs. Participants had to put five scenarios (ranging from ‘the physician decides everything alone’ to ‘I decide everything alone’) in order of their preferences.

In Study 2, *motives for the decision* (exploratory analysis) were measured with 17 items. Eleven items were taken from Bientzle et al,^41^ and six items were newly created. Participants evaluated the personal importance of the presented items on a seven‐point scale, where ‘1’ indicated low and ‘7’ indicated high importance. The contraception‐related motives assessed in this study were effectiveness (two items), costs (two items), well‐being, side‐effects, ease of administration (two items), long‐term usage, concerns about hormones, concerns about the implant of a foreign device, flexibility of administration, ease of becoming pregnant after end of use (two items), positive side‐effects, familiarity with the method, and control over the effectiveness. The motives were measured at both POMs.

In addition to the dependent variables, we examined potential *prior differences* among the experimental conditions. Participants in Study 1 were asked for age, gender, treatment preference, frequency of physical activity and prior experiences with knee injuries. In Study 2, they were asked for age, preference, duration of pill use, desire to have children and prior experiences with the copper chain.

As a *control question*, in Study 2 participants were asked at the end of the survey whether they had read a patient testimonial, a list with decision strategies, or a text about potatoes.

The similarities and differences between Study 1 and Study 2 are presented in Table 1.

**Table 1:** Similarities and differences between Study 1 and Study 2

	Study 1	Study 2
Hypothetical scenario	Cruciate ligament rupture (surgery or physiotherapy)	Contraceptive method (oral pill or IUD)
Content of decision aid	Consultation video + Information about preference‐sensitivity	Consultation video + Information about personal motives
Manipulation	Format of the additional material: Patient testimonial (narrative) Decision strategies c) Control group	Format of the additional material: Patient testimonial (narrative) Decision strategies c) Control group
Points of measurement	3 (pre [POM1], after video [POM2], after additional material [POM3])	2 (only pre [POM1] and after additional material [POM2])
Measures (DVs)	POM3: Preparation for decision making POM3: Evaluation of the decision POM1,2,3: Certainty and satisfaction POM1,2,3: Control preferences	POM2: Preparation for decision making POM2: Evaluation of the decision POM1,2: Certainty and satisfaction POM1,2: Motives (exploratory)
Data collection	Laboratory, at the computer	Online, at the computer

### 2.5 Statistical analysis

Data analysis of both studies was performed using the open‐source software R^55^ (R‐Packages^56^, ^57^, ^58^, ^59^, ^60^, ^61^). To test for differences among the conditions, we performed analyses of variance (ANOVAs) and mixed multivariate analyses of variance (MANOVAs) for the different POMs, combined with Bonferroni‐corrected post hoc tests. We pre‐registered only ANOVAs and contrast analysis. However, to reduce alpha error accumulation, we decided to use MANOVAs where possible and additionally used Bonferroni corrections. Normality distribution of the variables was checked using quantile plots, homogeneity assumptions of variance were tested using Levene's tests, and to test for the homogeneity of covariance matrices of the two dependent variables which were measured repeatedly, we computed Box's tests. For non‐parametric data, Kruskal‐Wallis tests were performed. We report all data as means (*M*) and standard deviations (SD). The level of significance was set at *P *< .05. Partial eta‐squared and Cohen's *d* were calculated as effect sizes of mean differences.

## 3 RESULTS

### 3.1 Prior analyses

In Study 1, no tested variable violated the assumption of normality or variance homogeneity (all *P ≥ *.069), and the repeated measurements did not violate the assumption of homogeneity of covariance matrices (*P = *.679). There were no group differences in Study 1 regarding age, gender distribution, treatment preferences, prior experiences with knee injuries or physical activity (all *P ≥ *.071). In Study 2, there were also no violations of the homogeneity of variances (all *P *≥ .094), and there was no violation of the assumption of homogeneity of covariance matrices (*P *= .145) among the repeated measurements. For the preparation for decision making, the quantile plot revealed a non‐normal distribution, but as previous literature has shown, the ANOVA is still robust even in the case of non‐normal distributions.^62^, ^63^ The groups in Study 2 also did not differ in terms of age, preference, duration of pill use, desire to have children and prior experiences with the copper chain (all *P ≥ *.181). Table 2 shows the means and standard deviations for the dependent variables in Study 1, Table 3 those for the dependent variables in Study 2.

**Table 2:** Means and standard deviations for the dependent variables by condition

	Narrative		Non‐narrative		Control		Overall	
	*M*	SD	*M*	SD	*M*	SD	*M*	SD
Preparation for decision making	4.00	1.42	4.26	1.23	1.68	1.39	3.31	1.77
Certainty								
POM1	3.83	1.90	3.69	1.65	3.59	2.02	3.70	1.85
POM2	4.85	1.56	4.21	1.69	4.46	1.82	4.51	1.70
POM3	5.14	1.61	4.50	1.74	4.32	1.83	4.65	1.75
Satisfaction								
POM1	4.59	1.56	4.29	1.12	4.07	1.58	4.32	1.44
POM2	5.45	1.17	4.92	1.26	5.21	1.27	5.20	1.24
POM3	5.61	1.20	5.11	1.22	5.29	1.22	5.34	1.22
Evaluation of the decision	5.52	0.99	5.41	1.04	4.86	0.91	5.07	0.83

**Table 3:** Means and standard deviations for the dependent variables by condition

	Narrative		Non‐narrative		Control		Overall	
	*M*	SD	*M*	SD	*M*	SD	*M*	SD
Preparation for decision making	4.96	1.19	5.06	1.16	2.07	1.62	4.10	1.88
Certainty								
POM1	4.99	1.91	4.90	1.85	4.10	1.74	4.67	1.84
POM2	5.27	1.98	4.67	1.81	4.03	1.72	4.67	1.82
Satisfaction								
POM1	5.06	1.48	4.95	1.59	4.22	1.54	4.75	1.58
POM2	5.61	1.38	5.39	1.47	4.50	1.42	5.17	1.50
Evaluation of the decision	5.52	0.99	5.41	1.04	4.86	0.91	5.33	0.89

### 3.2 Preparation for decision making

In Study 1, an ANOVA showed significant group differences in the feeling of being prepared for decision making, *F*(2, 107) = 40.63, *P *< .001, $\eta_{P}^{2}$ = 0.43. We conducted three Bonferroni‐corrected post hoc tests that supported Hypothesis 1a. Participants who had read either the narratives (*P *< .001, *d* = 1.65) or the decision strategies (*P *< .001, *d* = 1.96) felt better prepared for the decision than participants who had read the control text. Contrary to Hypothesis 1b, there was no significant difference between the narrative and the non‐narrative group, *P *= .430.

In Study 2, an ANOVA showed significant group differences in the feeling of being prepared for decision making, *F*(2, 214) = 140.33, *P *< .001, $\eta_{P}^{2}$ = 0.57. We conducted Bonferroni‐corrected post hoc tests that supported Hypothesis 1a. Participants who had read either the narratives (*P *< .001, *d* = 2.19) or the decision strategies (*P *< .001, *d* = 2.29) felt that the material had prepared them significantly better for the decision than participants who had read the control text. Contrary to Hypothesis 1b, there was no significant difference between the narrative and the non‐narrative group, *P *= .670.

### 3.3 Decision certainty and satisfaction

Contrary to Hypotheses 2a and 2b, a mixed MANOVA that tested for group differences over time revealed in Study 1 no significant interaction effects between condition and POM on decision certainty and satisfaction, *F*(8, 428) = 1.34, *P *= .222. There were no differences among the conditions in certainty and satisfaction, *F*(4, 214) = 0.97, *P *= .426, but significant changes over time, *F*(4, 428) = 16.62, $\eta_{P}^{2}$ = 0.05. Bonferroni‐corrected post hoc tests revealed a significant change in decision certainty, *P *< .001, $\eta_{P}^{2}$ = 0.05, and satisfaction over time, *P *< .001, $\eta_{P}^{2}$ = 0.11. This effect was due to a significant increase in decision certainty/ satisfaction from POM1 to POM2, *P *= .001, *d* = 0.46/ *P *< .001, *d* = 0.65, and from POM1 to POM3, *P *< .001, *d* = 0.53/ *P *< .001, *d* = 0.76, but not from POM2 to POM3, *P *= .530/ *P *= .420.

We computed a MANOVA to test for group differences over time on decision certainty and satisfaction with the decision in Study 2. Contrary to Hypotheses 2a and 2b, there was no significant interaction effect between group and POM, *F*(4, 428) = 1.61, *P *= .171, but there was a significant main effect of POM, *F*(2, 213) = 13.22, *P *< .001, $\eta_{P}^{2}$ = 0.04, and of group, *F*(4, 428) = 7.57, *P *< .001, $\eta_{P}^{2}$ = 0.05. Bonferroni‐corrected post hoc tests revealed that while decision certainty did not change between POM1 and POM2, *P *= .979, decision satisfaction changed from POM1 to POM2, *P *= .003, *d* = 0.29. There were also group differences in decision certainty/ satisfaction, *P *< .001, $\eta_{P}^{2}$ = 0.09/ *P *< .001, $\eta_{P}^{2}$ = 0.13. Participants in the control condition reported significantly lower decision certainty/ satisfaction with the decision than participants in the narrative group, *P *< .001, *d* = 0.75/*P *< .001, *d* = 0.86, and participants in the non‐narrative group, *P *= .002, *d* = 0.51/*P *< .001, *d* = 0.71. There were no significant differences in decision certainty/ satisfaction between the participants who had read the narratives or the decision strategies, *P *= .191/ *P *= .400.

### 3.4 Decision evaluation

Contradictory to Hypotheses 3a and 3b, there were no group differences in the evaluation of the decision in Study 1, *F*(2, 107) = 1.26, *P *= .289.

In Study 2, an ANOVA revealed significant group differences in the evaluation of the decision, *F*(2, 214) = 16.65, *P *< .001, $\eta_{P}^{2}$ = 0.13. Supporting Hypothesis 3a, participants who had read either the narratives, *P *< .001, *d* = 0.89, or the decision strategies, *P *< .001, *d* = 0.73, evaluated their decision more positively than participants who had read the control text. In contrast to Hypothesis 3b, there was no significant difference between the narrative and the non‐narrative group, *P *= .450.

### 3.5 Control preferences

Contradictory to Hypothesis 4, Kruskal‐Wallis tests showed no group differences regarding control preferences at any POM in Study 1, all *P *≥ .145.

### 3.6 Exploratory analysis of the effect on motives

In Study 2, the motives for making decisions about contraceptives the participants rated as most important were the effectiveness of the method, well‐being while using it, knowing the method, knowing one's tolerance for it, being able to control the effectiveness of the method, and little possibility of administration errors. To answer the open research question, whether DAs can support the participants in becoming more aware of what is personally important to them, we computed the individual absolute difference in the importance of each of the 17 items between POM1 and POM2. This measurement represented the individual change in importance of motives as a consequence of viewing the information material. A Welch test (Levene's test: *P *< .001) revealed a significant influence of the condition on the individual importance change, Welch's *F*(2, 2453.64) = 13.45, *P *< .001. Additionally performed *t* tests showed that individual importance change was significantly higher in the non‐narrative group (*M* = 0.77, SD = 1.03) than in the narrative group (*M* = 0.60, SD = 0.90), *t*(2450.50) = 4.33, *P *< .001, and the control group (*M* = 0.59, SD = 0.90), *t*(2440.60) = 4.80, *P *< .001. There was no significant difference between the narrative group and the control group, *t*(2428.50) = 0.52, *P *= .606.

## 4 DISCUSSION

In both studies, we found that participants who received additional material in the DA felt that the material prepared them better for the decision than participants in the control group. This effect is in line with the findings of Osaka and Nakayama^64^ and the assumptions of Elwyn et al.^26^ Decision certainty (in Study 1) and satisfaction (in both studies) were higher across all conditions after watching the consultation video. In Study 2, participants in the control condition reported lower decision certainty and lower satisfaction with the decision than participants in the narrative and non‐narrative groups. In Study 2, we also found that participants who had read either the narrative patient testimonials or the decision strategies evaluated their decision more positively than participants who had read the control text.

One possible explanation for the few effects in Study 1 could lie in the structure of our DA. The consultation video, which all participants watched before reading the testimonials or the decision strategies, showed a good consultation with an involved physician who explained the treatment options well and encouraged SDM. The finding that both decision certainty and satisfaction with the decision increased significantly between POM 1 and 2 indicates that participants perceived this part of the DA as very helpful for decision making. This finding supports the idea that a good understanding of the options, including risks and benefits, is essential in SDM^65^ and should be a minimum requirement for all DAs. The additional material was presented after the video, and as participants’ certainty and satisfaction were already quite high, there was little room to improve. We chose this procedure because we wanted to investigate the additional impact of our material. However, it may be more helpful to prepare patients for SDM before they receive information about the treatment options. The additional material was possibly less engaging, because it was presented in written form, while the consultation was presented as a video. Other studies have found that videos, compared to written texts, can lead to more satisfaction^20^, ^66^ and less uncertainty^67^ in patients.

Our participants in Study 1 were mostly university students and consequently relatively young and well‐educated. It is possible that younger and more educated people are more likely to expect that they can participate in medical decisions and need less encouragement to do so. Future studies aiming to evaluate additional material in DAs should consider varying the point at which they present such material (eg before or after receiving information about the options), the format (eg written or video) and the structure of their sample.

In Study 2, the change of individual importance regarding the motives between POM1 and POM2 was higher in the decision strategy group than in the other conditions. Listing disadvantages and advantages of the methods for each motive might have helped the participants to find out better what was personally important for them, whereas in the narratives the motives themselves might not have been described or explained clearly enough. The larger individual importance change for motives that were compatible with choosing the copper IUD can be explained by the new information participants received about the copper IUD. The participants had to evaluate motives they might have never thought about before, as the possible motives presented were not related to using the contraceptive pill. Future studies should take a more differentiated look at people's motives in medical decision making and how they are influenced by different kinds of information.

### 4.1 Limitations

There are limitations to our studies that need to be taken into consideration when interpreting the results. One point is that our decision situations were hypothetical and therefore maybe not personally relevant for the participants. Actual patients who suffer from a cruciate ligament rupture, for example, may think and feel differently from our participants and be more intensely involved. Also, we did not ask for prior experiences with medical treatments or the health system in general, and such prior experience could certainly influence medical decision making. In Study 2, the decision situation may have been easier than that in Study 1 to imagine, because all of the participants were young women who used a contraceptive method (ie the birth control pill). However, having already thought about other contraceptive methods was not a requirement for participation in our study, so the situation remained hypothetical for them. This choice to invite only women who were already taking the pill came along with some problems, because it restricted the generalizability of our results, and these women had already decided about a contraceptive method in the past.

We used a control text that was completely unrelated to any medical topic. This control text was not helpful in making any medical decision and therefore only served the purpose of finding out if our additional material was helpful at all, irrespective of the format used. Future studies could use different kinds of control texts, such as texts with related or unrelated medical information. Another problem may have been that we constructed the testimonials and the decision strategies ourselves. The material was not tested in a pilot study, and even though we received and implemented feedback from several members of our working group, we have no proof that the material included all of the aspects which may have been important or that it was as balanced as we intended.

Other possible narratives may have had a different impact on the decision‐making process. For example, we kept emotions at a minimum in our narratives, since our objective was to keep them comparable to the decision strategies. More emotional narratives may influence decision making in a different way. The consultation videos in both studies intended to show a good consultation with a physician who promoted SDM and who explained the treatment options in a comprehensible way. However, there may have been other factors that influenced the participants differently, like gender of the actors, or (in Study 2) how well they related to the patient in the video.

While the two studies were similar in many aspects, they also had important differences which make it difficult to compare their results. Study 1 took place in the laboratory, had three POMs, and the decision situation was purely hypothetical for the participants. Study 2 took place online, had only two POMs, and the participants were faced with a decision that was closer to their real‐life situation, because they had all made a decision about a contraceptive method at some point in the past. Conducting Study 2 online had the disadvantages that many participants did not finish the survey and we had less control of how much attention participants paid to the video and the texts. In order to reduce this problem, we included a minimum processing time and a control question, but we cannot know to what extent participants focused on the survey.

### 4.2 Implications

The participants benefitted consistently from the DAs used in our studies. The DAs supported participants’ understanding of treatment options, including risks and benefits, in a comprehensible and insightful way. Moreover, participants benefitted from additional support regarding their own preferences or motives. For the design of DAs, it seems highly recommendable to have a mixture of well‐balanced treatment information as well as support for patients in reflecting on their own preferences. It could be beneficial for physicians to apply such DAs as additional support for patients in the decision‐making process, or at least as a help to patients in becoming aware of their own preferences and motives during medical consultations.

## CONFLICTS OF INTEREST

No conflicts of interest are declared.

## AUTHORS’ CONTRIBUTION

All authors contributed substantially to the conception and design of this work. SK was involved in the acquisition of data. SK, ME and MB analysed and interpreted the data; all the authors contributed significantly to the discussion. JK, MB and ME drafted the manuscript; UC and SK commented on it and critically revised it. All of the authors approved to the final version to be published; all of the authors agreed to be accountable for all aspects of the work in ensuring that questions related to the accuracy or integrity of any part of the work would be appropriately investigated and resolved.

## ETHICAL APPROVAL

The research reported here was performed in accordance with the Declaration of Helsinki and had full approval by the local ethics committee (approval number: LEK 2019/016).

## TRIAL REGISTRY

The studies were pre‐registered on the pre‐registration platform AsPredicted (aspredicted.org) prior to data collection (Study 1:22.05.2019; registration number: #23822; http://aspredicted.org/blind.php?x=pi4gg7; Study 2:12.06.2019; registration number: #24710; http://aspredicted.org/blind.php?x=fh52f2).

## PATIENT OF PUBLIC CONTRIBUTION

There were no patients, service‐users or care‐givers involved in our study. The participants in our study were mostly university students who took part in an online experiment.

## Supporting information

Appendix S1

Appendix S2

Appendix S3

Appendix S4

Appendix S5

Appendix S6

Appendix S7

## Data Availability

The data sets of both studies and the corresponding scripts for the analyses are available from the following link: https://osf.io/6tvpk/.%201.^68^

## References

[1] Elwyn G , Dehlendorf C , Epstein RM , Marrin K , White J , Frosch DL . Shared decision making and motivational interviewing: achieving patient‐centered care across the spectrum of health care problems. Ann Fam Med. 2014;12(3):270‐275. 10.1370/afm.1615.Accessed September 23, 2020. 24821899 PMC4018376

[2] Müller‐Engelmann M , Keller H , Donner‐Banzhoff N , Krones T . Shared decision making in medicine: the influence of situational treatment factors. Patient Educ Couns. 2011;82(2):240‐246. 10.1016/j.pec.2010.04.028.Accessed September 23, 2020. 20542403

[3] O’Connor AMO , Llewellyn‐Thomas HA , Flood AB . Modifying unwarranted variations in health care: shared decision making using patient decision aids. Health Aff. 2004;23(S2): 10.1377/hlthaff.var.63. Accessed September 23, 2020. 15471770

[4] Elwyn G , Miron‐Shatz T . Deliberation before determination: the definition and evaluation of good decision making. Heal Expect. 2010;13(2):139‐147. 10.1111/j.1369-7625.2009.00572.x. Accessed September 23, 2020. PMC5060530 19740089

[5] Chewning B , Bylund CL , Shah B , Arora NK , Gueguen JA , Makoul G . Patient preferences for shared decisions: a systematic review. Patient Educ Couns. 2012;86(1):9‐18. 10.1016/j.pec.2011.02.004. Accessed September 23, 2020. 21474265 PMC4530615

[6] Elwyn G , Frosch D , Rollnick S . Dual equipoise shared decision making: definitions for decision and behaviour support interventions. Implement Sci. 2009;4(1): 10.1186/1748-5908-4-75. Accessed September 23, 2020. PMC2784743 19922647

[7] Kessler TM , Nachbur BH , Kessler W . Patients’ perception of preoperative information by interactive computer program ‐ exemplified by cholecystectomy. Patient Educ Couns. 2005;59(2):135‐140. 10.1016/j.pec.2004.10.009. Accessed September 23, 2020. 16257617

[8] Mira JJ , Tomás O , Virtudes‐Pérez M , Nebot C , Rodríguez‐Marín J . Predictors of patient satisfaction in surgery. Surgery. 2009;145(5):536‐541. 10.1016/j.surg.2009.01.012. Accessed September 23, 2020. 19375613

[9] Quaschning K , Körner M , Wirtz M . Analyzing the effects of shared decision‐making, empathy and team interaction on patient satisfaction and treatment acceptance in medical rehabilitation using a structural equation modeling approach. Patient Educ Couns. 2013;91(2):167‐175. 10.1016/j.pec.2012.12.007. Accessed September 23, 2020. 23318156

[10] Stiggelbout AM , Pieterse AH , De Haes JCJM . Shared decision making: concepts, evidence, and practice. Patient Educ Couns. 2015;98(10):1172‐1179. 10.1016/j.pec.2015.06.022. Accessed September 23, 2020. 26215573

[11] Wilson SR , Strub P , Buist AS , et al. Shared treatment decision making improves adherence and outcomes in poorly controlled asthma. Am J Respir Crit Care Med. 2010;181(6):566‐577. 10.1164/rccm.200906-0907OC. Accessed September 23, 2020. 20019345 PMC2841026

[12] Bagley CHM , Hunter AR , Bacarese‐Hamilton IA . Patients’ misunderstanding of common orthopaedic terminology: the need for clarity. Ann R Coll Surg Engl. 2011;93(5):401‐404. 10.1308/003588411X580179. Accessed September 23, 2020. 21943466 PMC3365461

[13] Weinman J , Yusuf G , Berks R , Rayner S , Petrie KJ . How accurate is patients’ anatomical knowledge: a cross‐sectional, questionnaire study of six patient groups and a general public sample. BMC Fam Pract. 2009;10:1‐6. 10.1186/1471-2296-10-43. Accessed September 23, 2020. 19523189 PMC2700077

[14] Sherlock A , Brownie S . Patients’ recollection and understanding of informed consent: a literature review. ANZ J Surg. 2014;84(4):207‐210. 10.1111/ans.12555. Accessed September 23, 2020. 24812707

[15] Elwyn G , O’Connor A , Stacey D , et al. Developing a quality criteria framework for patient decision aids: online international Delphi consensus process. Br Med J. 2006;333(7565): 10.1136/bmj.38926.629329.AE. Accessed September 23, 2020. PMC1553508 16908462

[16] Armstrong B , Spaniol J , Persaud N . Does exposure to simulated patient cases improve accuracy of clinicians’ predictive value estimates of diagnostic test results? A within‐subjects experiment at St Michael’s Hospital, Toronto. Canada. BMJ Open. 2018;8(2):1‐6. 10.1136/bmjopen-2017-019241. Accessed September 23, 2020. PMC5829891 29440215

[17] Armstrong B , Spaniol J . Experienced probabilities increase understanding of diagnostic test results in younger and older adults. Med Decis Mak. 2017;37(6):670‐679. 10.1177/0272989X17691954. Accessed September 23, 2020. 28199179

[18] Wegier P , Armstrong BA , Shaffer VA . Aiding risk information learning through simulated experience (ARISE): a comparison of the communication of screening test information in explicit and simulated experience formats. Med Decis Mak. 2019;39(3):196‐207. 10.1177/0272989X19832882. Accessed September 23, 2020. 30819033

[19] Stacey D , Légaré F , Lewis K , et al. Decision aids for people facing health treatment or screening decisions. Cochrane Database Syst Rev. 2017;4: 10.1002/14651858.cd001431.pub5. Accessed September 23, 2020. PMC6478132 28402085

[20] Bowers N , Eisenberg E , Montbriand J , Jaskolka J , Roche‐Nagle G . Using a multimedia presentation to improve patient understanding and satisfaction with informed consent for minimally invasive vascular procedures. Surgeon. 2015;15(1):7‐11. 10.1016/j.surge.2015.09.001. Accessed September 23, 2020. 26464072

[21] Knops AM , Legemate DA , Goossens A , Bossouyt PMM , Ubbink DT . Decision aids for patients facing a surgical treatment decision: a systematic review and meta‐analysis. Ann Surg. 2013;257:860‐866. 23470574 10.1097/SLA.0b013e3182864fd6

[22] Joseph‐Williams N , Elwyn G , Edwards A . Knowledge is not power for patients: a systematic review and thematic synthesis of patient‐reported barriers and facilitators to shared decision making. Patient Educ Couns. 2014;94(3):291‐309. 10.1016/j.pec.2013.10.031. Accessed September 23, 2020. 24305642

[23] Tobiano G , Bucknall T , Marshall A , Guinane J , Chaboyer W . Patients’ perceptions of participation in nursing care on medical wards. Scand J Caring Sci. 2016;30(2):260‐270. 10.1111/scs.12237. Accessed September 23, 2020. 26036723

[24] Joseph‐Williams N , Edwards A , Elwyn G . Power imbalance prevents shared decision making. BMJ. 2014;348(May):1‐3. 10.1136/bmj.g3178. Accessed September 23, 2020. 25134115

[25] Waldron T , Carr T , McMullen L , et al. Development of a program theory for shared decision‐making: a realist synthesis. BMC Health Serv Res. 2020;20(1):1‐17. 10.1186/s12913-019-4649-1. Accessed September 23, 2020. PMC6979294 31973754

[26] Elwyn G , Frosch D , Volandes AE , Edwards A , Montori VM . Investing in deliberation: a definition and classification of decision support interventions for people facing difficult health decisions. Med Decis Mak. 2010;30(6):701‐711. 10.1177/0272989X10386231. Accessed September 23, 2020. 21088131

[27] Eggeling M , Meinhardt A‐L , Cress U , Kimmerle J , Bientzle M .The impact of a physician’s recommendation and gender on informed decision making: A randomized controlled study in a simulated decision situation. *Heal Expect*. in press. 10.1111/hex.13161 PMC8077152 33274816

[28] Eggeling M , Bientzle M , Cress U , Shiozawa T , Kimmerle J . The impact of physicians’ recommendations on treatment preference and attitudes: a randomized controlled experiment on shared decision‐making. Psychol Heal Med. 2020;25(3):259‐269. 10.1080/13548506.2019.1687917. Accessed September 23, 2020. 31707838

[29] Shaffer VA , Hulsey L , Zikmund‐Fisher BJ . The effects of process‐focused versus experience‐focused narratives in a breast cancer treatment decision task. Patient Educ Couns. 2013;93(2):255‐264. 10.1016/j.pec.2013.07.013. Accessed September 23, 2020. 23962673

[30] Shaffer VA , Zikmund‐Fisher BJ . All stories are not alike: a purpose‐, content‐, and valence‐based taxonomy of patient narratives in decision aids. Med Decis Mak. 2013;33(1):4‐13. 10.1177/0272989X12463266 23065418

[31] Ubel PA . Is information always a good thing? Helping patients make “good” decisions. Med Care. 2002;40(9 Suppl):V39‐V44. 10.1097/01.MLR.0000023954.85887.69 12226584

[32] Entwistle AV , France EF , Wyke S , et al. How information about other people’s personal experiences can help with healthcare decision‐making: a qualitative study. Patient Educ Couns. 2011;85(3):e291‐298. 10.1016/j.pec.2011.05.014 21652162

[33] Bekker HL , Winterbottom AE , Butow P , et al. Do personal stories make patient decision aids more effective? A critical review of theory and evidence. BMC Med Inform Decis Mak. 2013;13(Suppl 2):S9. 10.1186/1472-6947-13-S2-S9 24625283 PMC4044102

[34] Mazor KM , Baril J , Dugan E , Spencer F , Burgwinkle P , Gurwitz JH . Patient education about anticoagulant medication: is narrative evidence or statistical evidence more effective? Patient Educ Couns. 2007;69(1‐3):145‐157. 10.1016/j.pec.2007.08.010 17942268

[35] Betsch C , Ulshöfer C , Renkewitz F , Betsch T . The influence of narrative v. statistical information on perceiving vaccination risks. Med Decis Mak. 2011;31(5):742‐753. 10.1177/0272989X11400419 21447730

[36] Winterbottom A , Bekker HL , Conner M , Mooney A . Does narrative information bias individual’s decision making? A systematic review. Soc Sci Med. 2008;67(12):2079‐2088. 10.1016/j.socscimed.2008.09.037 18951673

[37] Winterbottom AE , Bekker HL , Conner M , Mooney AF . Patient stories about their dialysis experience biases others’ choices regardless of doctor’s advice: an experimental study. Nephrol Dial Transplant. 2012;27(1):325‐331. 10.1093/ndt/gfr266 21642512

[38] Shaffer VA , Focella ES , Scherer LD , Zikmund‐Fisher BJ . Debiasing affective forecasting errors with targeted, but not representative, experience narratives. Patient Educ Couns. 2016;99(10):1611‐1619. 10.1016/j.pec.2016.04.004 27090559

[39] Monk AP , Davies LJ , Hopewell S , Harris K , Beard DJ , Price AJ . Surgical versus conservative interventions for treating anterior cruciate ligament injuries. Cochrane Database Syst Rev. 2016;(4):CD011166. 27039329 10.1002/14651858.CD011166.pub2 PMC6464826

[40] Streich NA , Zimmermann D , Bode G , Schmitt H . Reconstructive versus non‐reconstructive treatment of anterior cruciate ligament insufficiency. A retrospective matched‐pair long‐term follow‐up. Int Orthop. 2011;35(4):607‐613. 10.1007/s00264-010-1174-6 21127860 PMC3066313

[41] Bientzle M , Fissler T , Cress U , Kimmerle J . The impact of physicians’ communication styles on evaluation of physicians and information processing: a randomized study with simulated video consultations on contraception with an intrauterine device. Heal Expect. 2017;20(5):845‐851. 10.1111/hex.12521 PMC5600216 27860037

[42] Beer A , Lehermayr K , Bürger B , Brandt CJ .Wie sicher ist meine Verhütungsmethode? 2016. https://www.netdoktor.at/sex/verhuetung/sicherste‐verhuetungsmethode‐5463.

[43] Heßling A , Jugendsexualität BH . Jugendsexualität 2015: Die Perspektive Der 14‐ Bis 25‐Jährigen. Ergebnisse Einer Aktuellen Repräsentativen Wiederholungsbefragung. Köln: Bundeszentrale Für Gesundheitliche Aufklärung; 2015. www.bzga.de.

[44] Dehlendorf C , Grumbach K , Schmittdiel JA , Steinauer J . Shared decision making in contraceptive counseling. Contraception. 2017;95(5):452‐455. 10.1016/j.contraception.2016.12.010 28069491 PMC5466847

[45] Elwyn G , Frosch D , Thomson R , et al. Shared decision making: a model for clinical practice. J Gen Intern Med. 2012;27(10):1361‐1367. 10.1007/s11606-012-2077-6 22618581 PMC3445676

[46] Joseph‐Williams N , Elwyn G , Edwards A . Preparing patients ahead of time to share decisions about their health care. In: G Elwyn , A Edwards , R Thompson Shared Decision Making in Health Care: Achieving Evidence‐Based Patient Choice. 2016. Oxford: Oxford University Press, 43‐50.

[47] Shaffer VA , Tomek S , Hulsey L . The effect of narrative information in a publicly available patient decision aid for early‐stage breast cancer. Health Commun. 2014;29(1):64‐73. 10.1080/10410236.2012.717341 23384155

[48] Erwachsener V . Verhütungsverhalten Erwachsener 2018 (Bundeszentrale für gesundheitliche Aufklärung). https://www.forschung.sexualaufklaerung.de/verhuetung/verhuetungsverhalten‐2018/. Accessed September 23, 2020.

[49] Elwyn G , Durand MA , Song J , et al. A three‐talk model for shared decision making: multistage consultation process. BMJ. 2017;359:j4891. 10.1136/bmj.j4891 29109079 PMC5683042

[50] Wyatt KD , Anderson RT , Creedon D , et al. Women’s values in contraceptive choice: a systematic review of relevant attributes included in decision aids. BMC Womens Health. 2014;14(1): 10.1186/1472-6874-14-28 PMC3932035 24524562

[51] Bennett C , Graham ID , Kristjansson E , Kearing SA , Clay KF , O’Connor AM . Validation of a preparation for decision making scale. Patient Educ Couns. 2010;78(1):130‐133. 10.1016/j.pec.2009.05.012 19560303

[52] Stalmeier PFM , Roosmalen MS , Verhoef LCG , et al. The decision evaluation scales. Patient Educ Couns. 2005;57(3):286‐293. 10.1016/j.pec.2004.07.010 15893210

[53] O’Connor AM . Validation of a Decisional Conflict Scale. Med Decis Mak. 1995;15(1):25‐30. 10.1177/0272989X9501500105 7898294

[54] Degner LF , Sloan JA , Venkatesh P . The Control Preferences Scale. Can J Nurs Res. 1997;29(3):21‐43. 9505581

[55] R Core Team . R: A Language and Environment for Statistical Computing. Vienna: R Foundation for Statistical Computing; 2019. https://www.r‐project.org/.

[56] Fox J , Weisberg S An (R) Companion to Applied Regression. 3rd ed. Thousand Oaks, CA; 2019. https://socialsciences.mcmaster.ca/jfox/Books/Companion/.

[57] Torchiano M Effsize: efficient effect size computation. 2019. 10.5281/zenodo.1480624

[58] Kassambara A , rstatix: Pipe‐Friendly Framework for Basic Statistical Tests. R package version 0.6.0. 2020. https://cran.r‐project.org/package=rstatix

[59] Kassambara A . ggpubr: “ggplot2” Based Publication Ready Plots. R package version 0.4.0. 2020. https://cran.r‐project.org/package=ggpubr

[60] Fox J , Friendly M , Monette G .heplots: Visualizing Tests in Multivariate Linear Models. R package version 1.3‐5. 2018. https://cran.r‐project.org/package=heplots

[61] Revelle W . psych: Procedures for Personality and Psychological Research. 2018. https://cran.r‐project.org/package=psychVersion=1.8.12

[62] Blanca M , Alarcón R , Arnau J , Bono R , Bendayan R . Non‐normal data: is ANOVA still a valid option? Psicothema. 2017;29(4):552‐557. 10.7334/psicothema2016.38 29048317

[63] Schmider E , Ziegler M , Danay E , Beyer L , Bühner M . Is It Really Robust?: reinvestigating the robustness of ANOVA against violations of the normal distribution assumption. Methodology. 2010;6(4):147‐151. 10.1027/1614-2241/a000016

[64] Osaka W , Nakayama K . Effect of a decision aid with patient narratives in reducing decisional conflict in choice for surgery among early‐stage breast cancer patients: a three‐arm randomized controlled trial. Patient Educ Couns. 2017;100(3):550‐562. 10.1016/j.pec.2016.09.011 28277290

[65] Zeuner R , Frosch DL , Kuzemchak MD , Politi MC . Physicians’ perceptions of shared decision‐making behaviours: a qualitative study demonstrating the continued chasm between aspirations and clinical practice. Heal Expect. 2015;18(6):2465‐2476. 10.1111/hex.12216 PMC5810629 24938120

[66] Cornoiu A , Beischer AD , Donnan L , Graves S , De Steiger R . Multimedia patient education to assist the informed consent process for knee arthroscopy. ANZ J Surg. 2011;81(3):176‐180. 10.1111/j.1445-2197.2010.05487.x 21342392

[67] Chiou C‐P , Chung Y‐C . Effectiveness of multimedia interactive patient education on knowledge, uncertainty and decision‐making in patients with end‐stage renal disease. J Clin Nurs. 2012;21(9‐10):1223‐1231. 10.1111/j.1365-2702.2011.03793.x 21883569

[68] Eggeling M , Korger S , Cress U , Kimmerle J , Bientzle M .Data for paper: Decision aids to prepare patients for shared decision making: Two randomized controlled experiments on the impact of awareness of preference‐sensitivity and personal motives. https://osf.io/6tvpk/ 10.1111/hex.13159 PMC8077165 33517579

