# Supplementary material for: Decision aids to prepare patients for shared decision making: Two randomized controlled experiments on the impact of awareness of preference‐sensitivity and personal motives
Source: Health Expect. 2021 Jan 31;24(2):257–68. doi: 10.1111/hex.13159 (PMC8077165; doi:10.1111/hex.13159)
Supplement: Supplementary file 1 — Appendix S1 [file HEX-24-257-s001.docx]

**Appendix A**

Hypothetical scenario Study 1 (Cruciate ligament rupture)

**Imagine you are in the following situation:**

In your free time you like to do sports. A few weeks ago, you had an accident while playing sports: an inaccurate movement caused you to get your left knee twisted. At first you were in great pain, which became a little better due to cooling. Your knee has become very swollen since then and you can hardly walk. You went to your doctor to tell of your injury and the symptoms that you have had since then. The doctor examines your knee externally and tells you that the injury could potentially be a rupture of the anterior cruciate ligament. However, it is not yet possible to say exactly, as the knee is very swollen. Therefore, the doctor sends you to a radiologist to perform an MRI scan and clarify if your cruciate ligament is torn. In the meantime, you should take care of your knee and were given crutches to help.

Your doctor gives you a brochure to take home, which contains information on the cruciate ligament and cruciate ligament injuries.

**Please read the following information carefully.**

Hypothetical scenario Study 2 (Contraceptive method)

**Imagine you are in the following situation:**

Lately, you have been thinking about an alternative method of contraception to the one you use now. You have heard about the "copper chain”, an intrauterine contraceptive method, from a friend and now you want to ask your gynecologist for advice about this method.

Therefore, you have already made an appointment, which will take place in a few minutes. You have just entered your gynecologist’s practice, registered and now you are sitting in the waiting room.

In the waiting room you look at a brochure with information about the reliability of different contraceptive methods.

**Please read the following information carefully.**

***(at this point participants read the information text, see Appendix B)***

In the meantime, you have been called by the doctor's assistant and go to your gynecologist’s treatment room to get advice from her about the copper chain.
