# Supplementary material for: Decision aids to prepare patients for shared decision making: Two randomized controlled experiments on the impact of awareness of preference‐sensitivity and personal motives
Source: Health Expect. 2021 Jan 31;24(2):257–68. doi: 10.1111/hex.13159 (PMC8077165; doi:10.1111/hex.13159)
Supplement: Supplementary file 2 — Appendix S2 [file HEX-24-257-s003.docx]

**Appendix B**

General information text Study 1

What is the cruciate ligament and what do we need it for?

| The knee joint is stabilized by a number of ligaments. The cruciate ligament is part of this ligamentous apparatus. Without the cruciate ligaments, the knee would be very unstable. There are anterior and posterior cruciate ligaments, which intersect in the center of the joint - hence the name "cruciate ligament" (derived from the Latin “crux”, meaning cross). They connect thighs and shins and guide the knee in every movement. | 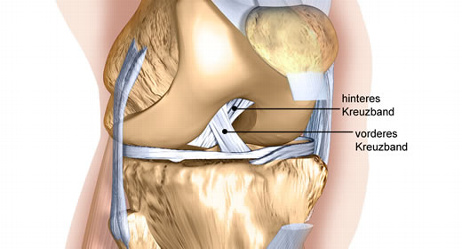 |
| --- | --- |

As you can see on the picture, the cruciate ligaments are deep in the knee joint. Therefore, injuries to the cruciate ligaments are difficult for doctors to recognize and often can only be detected with the help of magnetic resonance imaging (MRI). Both cruciate ligaments ensure that the knee remains stable when stretched and flexed and are responsible for controlling the rotation of the joint. Regardless of the joint position, at least parts of the cruciate ligaments are tightened. This demonstrates their important function in the stabilization of the knee joint.

How does the cruciate ligament get damaged?

| Despite their high resilience, the cruciate ligaments are susceptible to injuries. Rupture of the cruciate ligament is one of the most common and serious sports injuries, with more than 90% of cases affecting the anterior cruciate ligament. There is an especially great danger during skiing as well as ball sports like football or basketball. The anterior cruciate ligament tear is often a sports injury, which occurs by twisting the lower leg away from its natural mechanical axis (= sprain). | 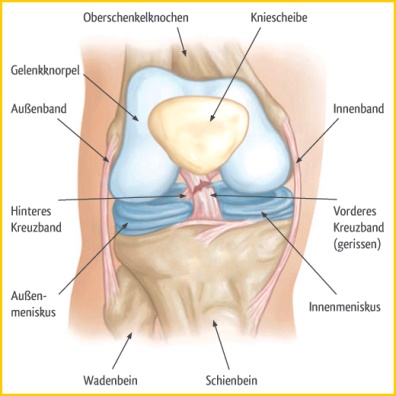 |
| --- | --- |

A typical injury situation is for example an attack against the leg of an opponent in football or a fall with twisting and/or hyperextension of the lower leg. This rotational movement puts increasing strain on the ligament, with too much stress causing a tear.

| 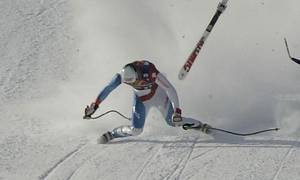 | 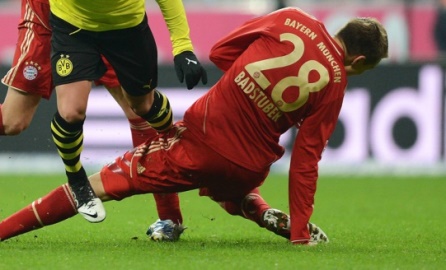 |
| --- | --- |

What does a cruciate ligament injury look like?

Most sufferers of a cruciate ligament injury feel a sharp, stabbing pain and some hear a crack at the moment of the tear. Typical symptoms include swelling of the knee joint, instability of the knee, problems with stretching and bending the knee, and bruising in the knee area. The severity of these symptoms and the associated pain can vary significantly.

| Recognizing a cruciate ligament tear is not easy for doctors, as the cruciate ligaments lie deep in the knee joint. Thus, it often happens that a diagnosis takes weeks or even years. Magnetic resonance imaging (MRI) or arthroscopy usually make it possible to diagnoses the injury. | 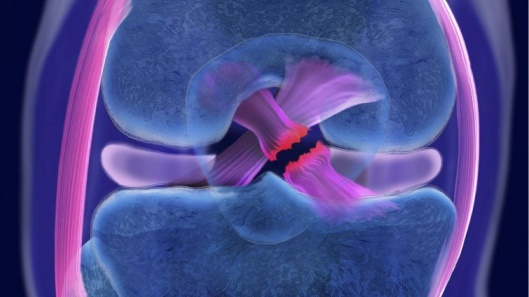 |
| --- | --- |

Swelling of the joint often occurs as a consequence of a cruciate ligament rupture, because during the accident blood vessels are also injured and this leads to bleeding in the knee joint. Due to this swelling, the instability of the knee is often not noticed at first. After the swelling subsides, sufferers often feel a wobbly feeling in the knee or feel the knee “give way.” A further consequence of a cruciate ligament tear, which can develop later on, is a type of knee joint wear, known as osteoarthritis.

Treatment options

After a tear of the anterior cruciate ligament there is the possibility to perform an operation in which the injured cruciate ligament is replaced by a transplant, or to treat conservatively, i.e. to strengthen the knee with the help of physiotherapy. Neither option is clearly preferable given the current scientific situation and both methods can lead to good treatment outcomes (Meuffels et al., 2009; Streich et al., 2011; Monk et al., 2016).

Regarding fitness for sport, a number of studies (e. g. Frobell et al., 2013; Lai et al., 2018; Myklebust et al., 2003) show that patients who undergo surgery can be active again at a similar level as before the accident in 23-83% of cases; patients who opt for a conservative method in 20-82%.
