# Supplementary material for: Decision aids to prepare patients for shared decision making: Two randomized controlled experiments on the impact of awareness of preference‐sensitivity and personal motives
Source: Health Expect. 2021 Jan 31;24(2):257–68. doi: 10.1111/hex.13159 (PMC8077165; doi:10.1111/hex.13159)
Supplement: Supplementary file 3 — Appendix S3 [file HEX-24-257-s005.docx]

**Appendix C**

***General information on the copper chain***

In addition to the contraceptive pill, this study will also look at the hormone-free contraceptive method "copper chain". The copper chain is an intrauterine ("inside the womb") long-term contraceptive. It consists of a surgical thread with copper tubes and is implanted in the uterine muscles at the upper node of the thread (see illustration).

***Text on the pearl index***

How safe are the contraceptive methods in comparison?

The Pearl Index, named after the American scientist Raymond Pearl, is the measure of contraceptive effectiveness. It indicates how many out of every 100 women who use the same contraceptive method become pregnant within a year ($Pearl-Index= \frac{Number of pregnancies*1200}{Number of women*Months of exposure}$). A Pearl index of 0.1 for a given contraceptive indicates that one in every 1000 women who use this contraceptive for more than 12 months will become pregnant. The Pearl index is sometimes calculated with and sometimes without taking into account application errors and is therefore subject to fluctuations in the literature.

The Pearl index of different contraceptive methods:

- No contraception: 30-85

- Birth control pill: 0.1-0.9

- Copper chain: 0,1-0,5

- Condom: 0,6-12
