# Supplementary material for: Decision aids to prepare patients for shared decision making: Two randomized controlled experiments on the impact of awareness of preference‐sensitivity and personal motives
Source: Health Expect. 2021 Jan 31;24(2):257–68. doi: 10.1111/hex.13159 (PMC8077165; doi:10.1111/hex.13159)
Supplement: Supplementary file 4 — Appendix S4 [file HEX-24-257-s004.docx]

**Appendix D**

Video script for Study 1

Part 1:

“Good day! I'm told that your knee injury is getting better. I'm glad to hear that, of course. However, it was very good that you were at the MRI, because unfortunately I have to tell you, it has confirmed my suspicion that your front cruciate ligament is torn. Fortunately, there were no further injuries. You have to know, in the context of such cruciate ligament tears, it often happens that the surrounding structures are affected, for example, that the meniscus is then also damaged. Luckily, we could rule that out, so for you that is not the case. However, it is still true that your anterior cruciate ligament is torn. I can show you that again here in the picture.

Here you can see the anterior cruciate ligament and here the posterior cruciate ligament. The two bands overlap here in the middle, which is why they are also called cruciate ligament. And here it is relatively clear to see that the anterior cruciate ligament is torn.

Now that we have this clear diagnosis, naturally the question arises of how we can help you get better and how we can treat you. It is important to know that there are basically two treatment methods: a surgery and a non-surgical treatment.

During surgery, the injured anterior cruciate ligament is replaced by a graft. This means that the broken cruciate ligament is removed and replaced by another tendon from your own body. In non-surgical treatment, the knee is then strengthened by means of intensive physiotherapy, i.e. physical therapy. Neither option is clearly preferable given the current scientific situation. Both methods can lead to good treatment success, but both have disadvantages. The choice of one or the other method therefore depends on your personal preference. It's important that you think about what's important to you personally.

I will now tell you something about the two different treatment methods. First about the surgical treatment method. As I said, the damaged cruciate ligament is replaced by a graft in the surgical treatment method. This is done as part of a hospitalization, in a 1 - 1.5 hour surgery, which we would perform under general anesthesia. The procedure would be done as an arthroscopy. "Arthroscopy" means "joint inspection", you may know this from the term endoscopy. We would introduce a camera, a light source and the surgical instruments via three small incisions on the knee joint and then have a very good view of the camera and the exposure into the surgical field. By doing this only very locally and minimally invasively, further scars or any major other scars would be prevented.

Yes, as said, the anterior cruciate ligament is then replaced by a graft. This is done in such a way that we would replace it with a tendon from your own body, since the tendon of the semitendinosus muscle can be used for this. This is the thigh muscle and it goes from the upper thigh to the knee joint and we would then take the tendon that is not necessary for the function of the thigh and then make the corresponding transplant for the anterior cruciate ligament.

Theoretically, such a surgery can be done right after the accident, which you may know from competitive sports, where athletes are operated 24 hours later. However, this is not necessary, I must emphasize, and with you that option is no longer possible anyway, but later surgeries, such as would be possible for you, are the most common. After the surgery, it would take a few weeks before you can move your knee normally again. We would, so to speak, promote the healing with supplementary measures, follow up through physiotherapy, in order to also provide support for the muscles and to restore the normal functioning of the knee as quickly as possible.

So now I would like to introduce you to the second treatment method, again, as I said this would be a non-surgical treatment in which the knee would undergo intensive physiotherapy training. This treatment is similar in principle to the postoperative rehabilitation that I have just introduced to you. In the beginning, it is primarily about counteracting pain and swelling and to minimize the loss of muscle in the knee area. You can imagine that by not moving your knee for a long time and not being able to move for a long time, the muscles in the surrounding area of the knee joint are breaking down. In the first step of physiotherapy, we would endeavor to counteract this loss so that you can bend and stretch your knee as soon as possible. In the second step, the focus is on muscle building and muscle activation. For this we would do an intensive strength and stability training and successively intensify the training period and the training treatment. And finally, in a third step, the protective reflexes would be trained in coordination exercises and in reactive training. So that, for example, if you take a surprising lunge, they can intercept it. And sports-specific movements, depending on what sports you do, will be practiced. Non-surgical treatment is therefore about building up gradual muscles around the knee joint and then compensating for the function of the failed anterior cruciate ligament with the help of these built-up muscles. So, you can imagine that we then target muscle building, which increases the stability of the knee joint in this area.

A non-surgical treatment we would start relatively early, because as I already told you, we would work directly to counteract pain and swelling and then just gradually continue in the therapy. If this therapy proves to be insufficient, the option to get the surgery will remain available in the future as well.

As I said, both methods of treatment have achieved equally good results, so you should think about which method to choose, which method makes more sense to you, which method you feel more comfortable with, and then we will follow that method.
