# Supplementary material for: Decision aids to prepare patients for shared decision making: Two randomized controlled experiments on the impact of awareness of preference‐sensitivity and personal motives
Source: Health Expect. 2021 Jan 31;24(2):257–68. doi: 10.1111/hex.13159 (PMC8077165; doi:10.1111/hex.13159)
Supplement: Supplementary file 5 — Appendix S5 [file HEX-24-257-s002.docx]

**Appendix E**

Video script for Study 2

**Scene 1: Greeting**

Patient opens the door and enters the consulting room, doctor stands from her desk. Both meet shortly before the doctor's desk and shake hands.

Doctor: "Good day Mrs. Bergmann."

Patient: "Good day."

Doctor points with her right hand to the free chair in front of her desk.

Doctor: "Please sit down."

Patient: "Thank you."

Patient sits on the chair in front of her desk, doctor sits on the chair behind her desk.

**Scene 2: Patient’s concern (clarity)**

Doctor: "What can I do for you?"

Patient: "Yes, well, in my circle of friends a good friend of mine has now decided to have the copper chain inserted …“

Doctor: "Mhm."

Patient: "... and she is very satisfied with it. She says she feels much better than with the pill and now I'm here to get information and to see if that might be something for me too."

Doctor: "Yes, the copper chain, we use it almost daily in my practice. So, I can imagine that I can help you."

Patient: "Very nice."

Doctor: "How are you currently practicing birth control?"

**Scene 3: “Patient forgets pill” (Reliability)**

Patient: "I am currently taking the pill ... “

Doctor: "Mhm."

Patient: "... and sometimes, when I have a lot on my mind, it can happen that I can't remember in the evening whether I took it in the morning or not, and then when I'm with my boyfriend and we want to have sex, I’m very insecure."

Doctor: "Well, of course it is not really ideal and understandable that you want to look for another alternative to the pill."

Patient: "Mhm."

Doctor: "Mhm. So, with the copper chain you don't have to worry about that anymore. Application mistakes are almost impossible once it has been inserted. And this is exactly why the copper chain is even safer than the pill is."

Patient: "Ah yes, I didn't know that."

Doctor: "Mhm."

Doctor starts turning around to look in her desk for a picture of the copper chain.

Doctor: "And wait a minute."

Searches for the picture in her desk and turns back to the patient.

Doctor: "Take a look."

Places the image on your desk. Patient leans forward to look at the picture.

Doctor: "This is what the copper chain looks like when it is inserted and if you want, I can give you some more information about the copper chain, so that you then can decide for yourself whether or not this is right for you".

Patient: "Yes, gladly."

Doctor: "Mhm. The copper chain was developed in the 80s and is a long-term contraceptive, which means it can stay three to five years in the uterus and will only then be replaced. It is also an emergency contraceptive, that is, if you have forgotten to take the pill, it is a an alternative to the 'morning-after pill' and, yes, particularly suitable is the copper chain if you live in a permanent partnership. If you change sexual partners frequently it is not quite as recommended, simply because it increased the risk of Cervical inflammation."

Patient: "Okay, that would not be the case with me."

Doctor: "Mhm, okay, good."

**Scene 4: “Foreign devide in the uturus” (Health/Wellbeing)**

Patient: "Um, yes, and that would be a real foreign body, which would then be placed in my uterus, right?"

Doctor: "Yes, and many women find the idea uncomfortable at first, but you really do not have to worry about that, because the physical risks of a copper chain are really very, very low, especially young women accept the copper chain much better than other contraceptives inserted into the uterus. In less than one woman in a hundred, the copper chain is rejected. Of course, this would mean that you would no longer be protected of pregnancy by the copper chain. In less than five women in a hundred, the copper chain has to be removed prematurely due to heavier bleeding or pain. The risk of cervical meningitis increases in the first six weeks after the copper chain is inserted, but studies have shown that the risk then falls back to normal. If cervicitis does occur, in rare cases it can also lead to infertility, as a result."

**Scene 5: “Infertility through the pill” (Perspective to become pregnant)**

Patient: "Infertility - mhm.

Doctor: "Yes, but you don't have to worry about that, as I said, the percentage of women who use the copper chain and who then become infertile is very, very low.

Patient: "Okay, yes, but I am also concerned about that when I take the pill. My neighbor is 31 and has always taken the pill and now she and her boyfriend have been trying to have a child for a long time and she has already had three miscarriages.

Doctor: "Mhm. You don't have to worry about that with the copper chain, because it only works locally. And if you decide to remove the copper chain, it will no longer affect your fertility from that point on.

Patient: "Ah, okay, that's comforting to hear."

Doctor: "Mhm."

**Scene 6: Goodbye**

Doctor: "Yes, do you have any other questions, Ms Bergmann?"

Patient: "Um, no, then I don't think I have any more questions. Then I will think about whether the copper chain is really for me.

Doctor: "Yes, I think this is a good way and I will make you the suggestion, if you have decided and would like to have the copper chain inserted, then contact me and otherwise we will see each other for the next preventive medical checkup.

Patient: "Okay, fine, yes then I'll do that. Thank you very much."

Doctor slips off the table in her chair. They both get up. Patient takes her handbag.

Doctor gives patient her hand.

Doctor: "Yes, my pleasure. Goodbye."

Patient: "Goodbye."

Doctor: "Bye."

Patient leaves the room. Doctor sits back down at her desk.
