# Supplementary material for: Decision aids to prepare patients for shared decision making: Two randomized controlled experiments on the impact of awareness of preference‐sensitivity and personal motives
Source: Health Expect. 2021 Jan 31;24(2):257–68. doi: 10.1111/hex.13159 (PMC8077165; doi:10.1111/hex.13159)
Supplement: Supplementary file 6 — Appendix S6 [file HEX-24-257-s006.docx]

**Appendix F**

Patient testimonials and decision strategies for Study 1

Testimonial 1 (female patient)

The diagnosis "cruciate ligament rupture" was a great shock for me. I was sure that my doctor would tell me which treatment he thought would be better, because in my experience that is what doctors almost always do. The fact that I, as a patient, should make a medical decision surprised me and at first made me feel insecure. I had to get used to the idea that surgery and physiotherapy really do work just as well!

I realized that in my eyes, somehow it was always the doctors who were the experts who had the medical knowledge and who therefore made the decisions. I thought that I couldn't make any meaningful decisions at all, as I lacked all the medical expertise that doctors have learned in their studies and in years of experience.

But then I realized that it is not just about expertise. My doctor explained to me really well how a surgery and a physiotherapy would work and what the advantages and disadvantages would be, but of course I didn't understand every detail. Then he said that I had to think about what my personal preferences were, i.e. what was important to me for the treatment. It's not easy, because it's a completely new situation, with which you have no experience, and the information from the doctor was quite abstract. I then tried to think about what surgery and physiotherapy would mean for me and my everyday life. I also talked about it with my friends. That helped and I quickly realized in the conversation that some things are more important to me than others.

Overall, I have learned that I can really take my wishes and worries seriously, as they are central to the decision to have a surgery or physiotherapy. Both are important: the doctor's expertise and my own needs and expectations. That was a good experience.

Testimonial 2 (male patient)

I was so used to being informed by the doctor, but not really involved in medical decisions. But it is obvious that this should be the case, after all, I know myself and my body best! Only I can feel what is really good for me, and a torn cruciate ligament in particular is a lengthy process, where treatment takes a long time and requires a lot of perseverance.

The doctor took the time to explain the options to me in detail and that was important because I didn't know anything about the cruciate ligament, the structure of the knee and the differences between surgery and physiotherapy in my situation. Of course, I had many questions and I asked them, so that in the end I really had a very good overview. It was particularly important for me to find out about all the possible risks, so that I could then assess for myself what I could handle more and what I could handle less. I think it's important to ask questions openly, because doctors sometimes don't know exactly what is difficult for us patients to understand. Besides, every patient is different.

As a patient, you're still not an expert, of course. That's why doctors study medicine, but I had understood enough to be able to imagine what surgery or physiotherapy would mean for me. That is important, because how else would you be able to decide? It helped me to do more research on the topic myself. I also imagined different situations that might happen during surgery or physiotherapy and then thought about how I would feel. Getting factual information is one thing, but in the end, I have to feel good about the decision and go through with it!

The decision-making process was not an easy one for me, but overall a good experience.

Decision strategies

In medical decision-making situations where there is more than one equally good treatment option, patients should be involved in the decision. In these situations, there are some strategies that can help you make a decision.

These strategies are:

- Get an overview of how the treatment options work and what their advantages and disadvantages are.
- Ask the doctor questions.
- Search for further information yourself.
- Exchange with others.
- Become aware that doctors are experts in medical knowledge, but that you yourself are an expert for your own needs and body.
- Imagine the different treatment options and consider how you would feel in the specific situation.
