# Supplementary material for: Decision aids to prepare patients for shared decision making: Two randomized controlled experiments on the impact of awareness of preference‐sensitivity and personal motives
Source: Health Expect. 2021 Jan 31;24(2):257–68. doi: 10.1111/hex.13159 (PMC8077165; doi:10.1111/hex.13159)
Supplement: Supplementary file 7 — Appendix S7 [file HEX-24-257-s007.docx]

**Appendix G**

Patient testimonials and decision strategies for Study 2

Testimonial 1 (more positive toward copper chain):

After a good friend of mine told me about her positive experience with the copper chain, I also asked myself whether I should switch from the pill to the copper chain and I consulted my gynecologist. When she told me that neither the pill nor the copper chain was considered to be a clearly better method, I first had to consider what was important to me in contraception.

My side effects from the pill - such as mood swings, less desire for sex and the risk of thrombosis - and the feeling that I am adding extra hormones to my body have actually been worrying me for quite some time. Since I started studying and travelling more than before, it has also happened that I was not at home at the time I was supposed to take the pill. Consequently, it has also happened to me a few times that I forgot to take it.

With the copper chain you don't have this problem, because once it has been inserted correctly, application errors are no longer possible and at the same time it has a low Pearl index. With it I can simply have peace of mind for five years and no longer have to worry about contraception. Although the copper chain can cause pain or a heavier period, most women tolerate it so well that the chain can remain in place for five years. I don't want to have children in the near future, but when I do in a few years, my gynecologist can simply remove the copper chain and I can theoretically become pregnant immediately. So far, I have paid €40 for my 6-month pack of the pill. If I calculate the cost of the copper chain (350 €) like this, I even save 5 € every six months.

I would advise everyone to find out more about the subject. Since I know so much about contraceptive methods, I feel even more confident and have been able to make a decision that suits what is important to me personally.

Testimonial 2 (more positive toward contraceptive pill)

After taking the pill continuously for several years, I recently had a consultation with my gynecologist about contraceptive methods. This was the first time I realized how important my own opinion is when deciding on a contraceptive. I didn't even know that the copper chain was an equivalent alternative to the pill. I slowly started to get more familiar with the topic, and I realized what I really wanted in terms of contraception.

First of all, it is very important to me that my contraceptive method reliably protects me from pregnancy. The pill has a low Pearl index and by thinking about it every day, I can control myself that no mistakes are made when using it. With the pill, I already know what side effects I have to expect: I have gained some weight and occasionally had some bleeding, but the pill has made my menstrual pain and my skin better and if the side effects are too much for me, I can always switch to another pill.

With the copper chain, I am simply unsettled by the fact that a foreign device is inserted into the uterus, which probably hurts quite a lot, and the uterus can be pierced. The copper chain can also be expelled, or you can get an inflammation of the abdomen, especially in the beginning, which can even lead to infertility.

When I want to have children in a few years' time, I know that I can simply stop taking the pill and my hormone levels will settle down again - albeit after a while - so that I can get pregnant. I don't really want to go through this change before then, but I want to stick to my method for now. If, for example, I want to have children in three years' time, the cost of inserting the copper chain (which is probably between €250-450) would not be worth it.

Now that I have made another conscious decision, I feel even better about it, simply because I have learned a lot about how the pill works and what alternatives there are.

Decision strategies

Many women use the contraceptive pill and have to decide at some point whether to switch to a long-term device like the copper chain. As neither method of contraception is clearly better than the other, the decision depends solely on the patient's preferences and needs. For making this decision, it can be helpful to find out more about the pill and the copper chain and to consider what is important to you personally when using contraception.

In the following, we have therefore put together a list of different reasons for using one contraceptive method or another.

**Reasons that may play a role in the decision:**

**Pregnancy protection:** Both methods have a low Pearl Index and provide reliable protection against pregnancy.

**Familiarity with the method:** Women who use the pill already know how the pill works, how well they are coping with the method and what side effects they are experiencing with their pill, or they can also switch to another pill.

**Side effects:**

- - The pill has possible side effects, such as inter-bleeding, mood swings, weight changes, reduced libido, and an increased risk of thrombosis.
  - Possible side effects of the copper chain are a heavier period or pain from wearing the copper chain. In the first 20 days after the copper chain is inserted, there is an increased risk of pelvic inflammation, which can lead to tubal pregnancy or infertility. Ejection of the copper chain may occur. Pain may occur during insertion and the uterus may be pierced.

**Positive side effects of the method**

- - The pill can have positive effects on menstrual problems and the skin.
  - The copper chain can remain in use for up to five years.

**Costs:**

- - The pill costs about 40 € for six months.
  - The insertion of a copper chain costs about 350 € once (lasts up to five years, 35 € every six months).

**Application and possible application errors:**

- - The user can flexibly start and stop taking the pill. It is up to the user to make sure that she does not forget to take a pill (which would reduce the contraceptive protection); the pill should always be taken at the same time if possible.
  - The copper chain is inserted by a gynecologist (which may cause pain) and is removed by a gynecologist (after five years at the latest); if the copper chain is correctly positioned, errors in application are practically impossible.

**Intervention in the body:**

- - The use of the pill involves an intervention in the hormone balance.
  - With the copper chain a foreign device is inserted into the uterus.

**Prospect of pregnancy:**

- - After stopping the pill, the date of a possible pregnancy may be delayed by the renewed change in hormone balance
  - After removal of the copper chain, immediate pregnancy is generally possible.
